# Tinnitus-related distress and pain perceptions in patients with chronic tinnitus – Do psychological factors constitute a link?

**DOI:** 10.1371/journal.pone.0234807

**Published:** 2020-06-25

**Authors:** Benjamin Boecking, Josephine von Sass, Antonia Sieveking, Christina Schaefer, Petra Brueggemann, Matthias Rose, Birgit Mazurek

**Affiliations:** 1 Tinnitus Center, Charité–Universitätsmedizin Berlin, Berlin, Germany; 2 Division of Psychosomatic Medicine, Medical Department, Charité–Universitätsmedizin Berlin, Berlin, Germany; Universiteit Antwerpen, BELGIUM

## Abstract

**Objective:**

To investigate the co-occurrence of tinnitus-related distress and pain experiences alongside psychological factors that may underlie their association.

**Method:**

Patients with chronic tinnitus (*N* = 1238) completed a questionnaire battery examining tinnitus-related distress and affective and sensory pain perceptions. A series of simple, parallel- and serial multiple mediator models examined indirect effects of psychological comorbidities as well as -process variables including depressivity, perceived stress and coping attitudes. Moderator and moderated mediation analyses examined differential relational patterns in patients with decompensated vs. compensated tinnitus.

**Results:**

There were significant associations between tinnitus-related distress and pain perceptions. These were partially mediated by most specified variables. Psychological comorbidities appeared to influence tinnitus-pain associations through their impact on depressivity, perceived stress, and coping attitudes. Some specific differences in affective vs. sensory pain perception pathways emerged. Patients with decompensated tinnitus yielded significantly higher symptom burden across all measured indices. Tinnitus decompensation was associated with heightened associations between [1] tinnitus-related distress and pain perceptions, depressivity and negative coping attitudes; and [2] most psychological comorbidities and sensory, but not affective pain perception. Moderated mediation analyses revealed stronger indirect effects of depressivity and anxiety in mediating affective-, and anxiety in mediating sensory pain perception in patients with decompensated tinnitus.

**Conclusion:**

Psychological constructs mediate the co-occurrence of tinnitus- and pain-related symptoms across different levels of tinnitus-related distress. Psychological treatment approaches should conceptualize and address individualised interactions of common cognitive-emotional processes in addressing psychosomatic symptom clusters across syndromatic patients with varying distress levels.

## Introduction

Both chronic tinnitus and pain are subjective, multifactorially influenced sensations [1, 2]. Beyond potential sensory or neurological contributors [3, 4], cognitive-affective processes are known to play key roles in the subjective experience, maintenance and potential chronification of each syndrome [5, 6, 7]. A subgroup of people with chronic tinnitus–conceptualized as a phantom auditory perception [8]–experience considerable emotional distress [9, 10] and report high levels of depression [11], anxiety [12] and other somatoform symptoms [13, 14] constituting the phenomenon of “decompensated” (vs. “compensated”) tinnitus. In a more process-focused domain, those affected report high levels of perceived stress [15, 16, 17, 18, 19] and negative coping-related attitudes such as lowered optimism, self-efficacy beliefs, or heightened pessimism. These coping attitudes may affect tinnitus-related distress through their impact on general emotional distress [20, 21, 22]. Analogously, pain perceptions is frequently accompanied by high levels of emotional [23, 24, 25] or perceived stress [26], and a substantial body of work has highlighted interactions of cognitive and affective factors in mediating experiences of pain sensations [27, 28, 29, 30, 5, 31, 32, 33]. Consequently, tinnitus- or pain related distress are key targets of psychological or multimodal interventions that have been shown to be effective [13, 34, 35, 36]. However, despite the intriguing overlap between tinnitus-related distress and pain perceptions [3], hardly anything is known about their potential co-occurrence–or the role of psychological factors in mediating possible associations. Only one clinical study investigated the co-occurrence of pain perceptions and tinnitus and reported that 54.2% of *N* = 77 patients with pain perceptions also reported suffering from tinnitus [37]. Some other studies reported associations between tinnitus and headaches or migraines [38, 39, 40, 41] or temporomandibular joint pain [42, 43, 44]. However, these studies did not hypothesize or examine psychological factors such as psychological comorbidities, perceived stress, or coping attitudes as possible common denominators. Given the conceptual similarity of both symptom clusters, as well as the established importance of cognitive-emotional distress in contributing to the maintenance of either, the current study investigates tinnitus-related distress and pain perceptions in a sample of *N* = 1238 patients with chronic tinnitus. We hypothesized that both factors correlated and that psychological comorbidities would mediate respective associations. Exploratory analyses further examined whether [1] psychological comorbidities might exert their effects through their impact on individuals’ levels of depressivity, perceived stress and coping attitudes and [2] tinnitus decompensation, i.e. high levels of tinnitus-related distress[45], differentially influence relations between symptom-related and mediating factors. Specifically, we investigated the following hypotheses:

1. Tinnitus-related distress is significantly associated with affective and sensory pain perceptions. Each construct correlates positively with psychological comorbidities, depressivity, perceived stress, and pessimism and negatively with self-efficacy and optimism.
2. Psychological comorbidities, depressivity, perceived stress and coping attitudes mediate the relationships between tinnitus-related distress and affective or sensory pain perceptions.
3. [Exploratory]: Psychological comorbidities may exert such effects *through* their impact on depressivity, perceived stress and coping attitudes.
4. Compared to patients with compensated tinnitus, patients with decompensated tinnitus show significantly higher levels of symptom burden across indices of pain perception and putative mediators.
5. [Exploratory]: Compared to patients with compensated tinnitus, patients with decompensated tinnitus may show differences in relationships between [a] tinnitus-related distress and putative mediators, [b] putative mediators and pain perception, and [c] tinnitus-related distress and sensory and affective pain perceptions.
6. [Exploratory]: Indirect effects may differ for patients with decompensated vs. compensated tinnitus.

## Method

### Participants

The present study includes self-report data from *N* = 1238 patients who [a] self-referred to the Tinnitus Centre at Charité Universitätsmedizin Berlin between January 2011 and October 2015, [b] suffered from chronic tinnitus (lasting for > 3 months), [c] were 18 years of age or older and [d] completed both the Tinnitus Questionnaire and the Pain Perception Scale. Exclusion criteria comprised the presence of acute psychotic illness or addiction, (untreated) deafness and insufficient knowledge of the German language. The total dataset comprised *N* = 3851 patients with chronic tinnitus with equal gender proportions (47.1% female). Two-thousand-six-hundred-thirteen (*n* = 2613; 67.9%) datasets were excluded for containing missing values for the Pain Perception Scale (2585; 67.1%) and/or the Tinnitus Questionnaire. Potential–unrecorded–reasons may have included patient refusal, technical difficulties or fatigue effects (as the Pain Perception Scale featured last in the questionnaire dataset). Note that missing values for the Pain Perception Scale do *not* indicate the absence of pain experiences–which could be explicitly indicated in the scales’ ratings. Excluded cases were slightly, but significantly older than those included in the final sample (*M*_*exluded*_ = 51.22; *SD*_*exluded*_ = 13.49; *t*(3849) = -2.34, *p* = .02). Table 1 provides an overview of the sample’s sociodemographic characteristics. Upon arrival at the Tinnitus Centre, patients completed a routine questionnaire assessment battery on Acer Pocket PC n300 electronic handheld information devices. Participants provided written consent for data to be collected and used for research purposes, and the Charité Universitätsmedizin Berlin’s ethics committee approved data collection and analysis (No: EA1/040/08).

**Table 1:** Sociodemographic information (*N* = 1238 patients with chronic tinnitus).

Variable	*M*	*SD*
Age	50.17	12.02
	*n*	*%*
Gender		
Male	614	49.6
Female	624	50.4
Duration of tinnitus		
<1/2 year	159	12.8
1/2–1 year	252	20.4
1–2 years	188	15.2
2–5 years	216	17.4
>5 years	423	34.2
Degree		
None	37	3
Current: senior	9	0.7
Current: apprentice	8	0.6
Current: university	41	3.3
Apprenticeship	349	28.2
Polytechnic degree	193	15.6
University degree	601	48.5
Nationality		
German	1177	95.1
Other	61	4.9
Relationship status		
Single	382	30.9
Married	645	52.1
Divorced	188	15.2
Widowed	23	1.9
Work status		
Employed	902	72.9
Unemployed	336	27.1

*M* = mean, *SD* = standard deviation.

### Measures

#### Tinnitus-related distress

The German version of the tinnitus questionnaire [46] assesses the impact of tinnitus across various psychological dimensions. It consists of 52 statements that are answered on a 3-point scale (0 = *not true*, 1 = *partly true*; 2 = *true*) across five subscales (cognitive and emotional burden, persistence of sound, hearing difficulties, sleep difficulties, and somatic complaints). It has been suggested that only the total score should be interpreted [47]–a recommendation that is followed in this paper. The total score uses 40 items with two being included twice, thus yielding a score from zero to 84. Biesinger et al. [48] suggested a cut-off of 46 points to distinguish high vs. low symptom burden; i.e. denote decompensated vs. compensated tinnitus. The scale’s test-retest reliability is good (total score: *r* = 0.94; [49]). In the current sample, the scale’s internal consistency was excellent (α = 0.92).

#### Pain characteristics

Frequency and intensity of patients’ pain perceptions were measured using two visual-analogue scales anchored at 0 [*never*/*minimal*] and 10 [*permanently*/*maximal*].

#### Pain perception

The Pain Perception Scale *(“Schmerzempfindungsskala"”-SES;* [50]) measures subjective pain perceptions across an affective and sensory scale. The former comprises 14 items that inquire about subjective pain-related affective distress [general affective pain statement, persistence indication of pain] whilst the latter comprises 10 items that inquire about subjective descriptions of physically experienced pain sensations [rhythm, local intrusion, and temperature]. All items are answered on a 4-point-scale (1 = *does not apply*, 2 = *hardly applies*, 3 = *somewhat applies*, 4 = *completely applies*) with scores ranging from 14–56 [affective pain perception] and 10–40 respectively [sensory pain perception]. Relative to a clinical reference population of patients with pain perceptions, the affective pain perception scale yields cut-off ranges of < 22 (below average), 22–44 (average), and > 44 (above average). For the sensory pain perception scale, cut-off score-ranges are < 12, 12–25, and > 25. Notably, given the nature of reference sample, below-average values can also indicate considerable pain-related distress compared to the healthy general population [51]. In the present study, both types of pain perception were conceptualized as dimensionally distributed traits; however, category frequencies are reported descriptively. The scale’s test-retest reliability is good (*r* = 0.89–0.96) with internal consistency being moderate to high (α = 0.72–0.92; [50]). In the current sample, internal consistencies were excellent (α_affective_ = 0.96; α_seensory_ = 0.90).

#### Psychological comorbidities

Psychological comorbidities concomitant to the index symptom “chronic tinnitus" were measured using the ICD-10 Symptom Rating [52,53]. The ISR consists of 29 items that are answered on a 5-point-scale (0 = *does not apply*, 1 = *hardly applies*, 2 = *somewhat applies*, 3 = *considerably applies*, 4 = *completely applies*). The measure includes five subscales that measure the presence or severity of depressive, anxiety-related, obsessive-compulsive, somatoform [including health-anxiety] and eating-related symptoms which link to syndromatic diagnostic categories as defined in the International Classification of Diseases-10 [54]. A supplementary scale further measures additional indices of psychological distress, clinical relevance or specific syndromes. Indexing the extent of overall emotional impairment, a total score is calculated that weighs the supplementary scale twice. All indices are linearly transformed to range from 0 to 1. Cut-off scores are 0.5 (total score), 0.75 (depressive and anxiety-related syndromes), 0.67 (obsessive-compulsive syndrome), and 0.33 (somatoform and eating-related syndromes) [55]. Test-retest reliability is good (*r* = 0.84–0.84; [52]). In the current sample, internal consistency was excellent (α = 0.93).

#### Depressivity

Depressivity was measured using the German version of the Center for Epidemiological Studies Depression Scale (“Allgemeine Depressionsskala”-ADS; [56, 57]. The scale comprises 20 items that measure emotional, motivational, cognitive, somatic and motoric symptoms of low mood on a 4-point-Likert scale (0 = *rarely*, 1 = *sometimes*, 2 = *often*, 3 = *almost always*) yielding a range from 0 to 60. A cut-off score of 23 suggests major depressive disorder; however, the present study conceptualized depressivity as a dimensionally distributed trait [58,59]. Test-retest reliability is moderate (*r* = 0.51–0.67) with internal consistency ranging from 0.85 to 0.92 [57]. In the current sample, internal consistency was sufficient (α = 0.73).

#### Perceived stress

Subjectively perceived stress was measured using the Perceived Stress Questionnaire-PSQ [60,61]. The scale measures perceived stress across four dimensions three of which constitute facets of one’s internal stress reaction (tension, worries, [lack of] joy) and one of which measures perceived external stressors (demands). *Tension* explores tense disquietude, exhaustion and lack of relaxation. *Worries* assesses anxious concern for the future, and feelings of desperation and frustration; *joy* assesses positive feelings of challenge, joy, energy, and security and *demands* assesses perceived environmental demands such as lack of time, pressure, and overload. The scale consists of 30 items that are rated on a 4-point scale (1 = *almost never*, 2 = *sometimes*, 3 = *often*, 4 = *almost always*). All indices are linearly transformed to range from 0 to 1. All scores are subsumed in a total score for which *joy* is recoded. Whilst the present paper analyses perceived stress as a dimensional concept, suggested cut-off scores (defined as one *SD* > healthy population mean) are 0.50 (total score), 0.55 (tension), 0.46 (worries), <0.41 (joy), and 0.57 (demands) [61]. In the current sample, internal consistency was good (α = 0.90).

#### Coping attitudes

Adaptive and maladaptive coping attitudes were measured using the *Self-Efficacy-Optimism-Pessimism-Scale (“Selbstwirksamkeits-Optimismus-Pessimismus-Skala”-SWOP;* [62]). The scale comprises nine items that are answered on a 4-point scale (1 = *does not apply*, 2 = *hardly applies*, 3 = *somewhat applies*, 4 = *completely applies*) and load on three independent scales with mean scores ranging from 1 to 4: self-efficacy, optimism and pessimism. In the current sample, internal consistencies were sufficient (α_self-efficacy_ = 0.82; α_optimism_ = 0.79; α_pessimism_ = 0.65).

### Data analyses

**Fig 1:**
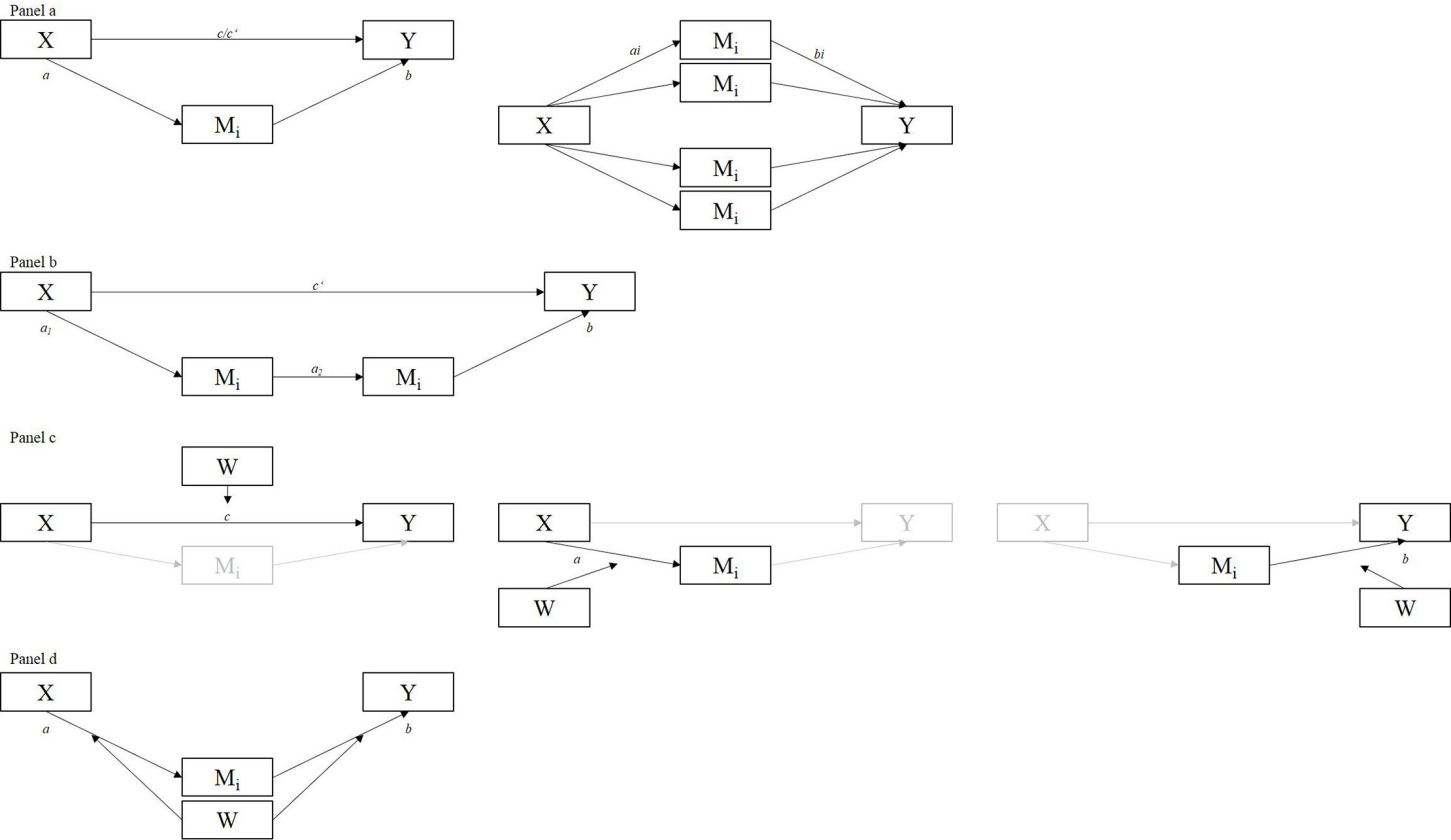
Conceptual diagrams of the specified models. *Panel a*: Simple and parallel multiple mediator models specifying tinnitus-related distress as independent, the putative mediators’ total (left) or subscale scores (right) as mediating, and affective or sensory pain perception as dependent variables. *Panel b*: Serial multiple mediator models specifying tinnitus-related distress as independent, psychological ‘comorbidities’ as first-level mediating variables, psychological process variables (depressivity, perceived stress, and coping attitudes) as second-level mediating variables, and affective or sensory pain perception as dependent variables. *Panel c*: Simple moderator models investigating the effect of tinnitus decompensation vs. compensation (W) on paths c (left), a (middle), or b (right). *Panel d*: Moderated mediation model investigating the effect of tinnitus decompensation vs. compensation on *ab*.

We used IBM SPSS Statistics for Windows, Version 24 to conduct the reported statistical analyses. Pearson’s correlation coefficient (*r*) examined the relationship between all measures. The visual analogue scales were split into quartiles for descriptive reports of patients scoring in each scale range. Crosstabulations investigated frequencies of patients scoring above vs. below cut-off scores across the pain perception and tinnitus-related distress scales. Comparisons of descriptives between patients with decompensated vs. compensated tinnitus were computed using univariate ANOVA. Effect sizes *d* were calculated separately [63] with estimates being defined as small (0.20–0.49), medium (0.50–0.79) or large (> 0.80; [64]). Moderator and mediator analyses were conducted using the *process* macro by Hayes [65]. Effects of the independent variable *X* on the dependent variable *Y* are denoted as *total effects c*; effects of X on the mediator *M* as paths *a*; and effects of M on Y as *b*. Indirect effects are denoted as *ab*; and the total effects adjusted for *ab* as *direct effects c’*. Whenever the effect of X on Y decreases significantly (but not to zero), upon consideration of *ab*, “partial mediation” occurs [66]. First, simple mediator models specified tinnitus-related distress as independent (X), the total scores of the candidate process variables as mediating (M_i_), and affective or sensory pain perception as dependent variables (Y). Follow-up analyses specified parallel multiple mediator models to investigate indirect effects via the PSQ and ISR’s subscale scores to account for the subscales’ intercorrelations whilst assuming non-causal associations (Fig 1, *Panel a*). Second, serial multiple mediator models explored whether psychological comorbidities exerted indirect effects *through* their impact on depressivity, perceived stress or coping attitudes. Here, tinnitus-related distress was specified as independent, psychological comorbidities (ISR) as first-step mediating, psychological process variables (ADS, PSQ, SWOP) as second-step mediating, and affective or sensory pain perceptions as dependent variables (*Panel b*). Third, to investigate differences in effects associated with tinnitus decompensation, path coefficients *c*, *a* and *b* were compared specifying decompensated vs compensated tinnitus severity as binary moderator *W* (*Panel c*). Finally, moderated mediation analyses tested whether potential indirect effects *ab* differed across categories of *W* (*Panel d*).

## Results

### Descriptive indices

Table 2 provides means and standard deviations for the total sample as well as descriptors of symptom levels where applicable. Overall, patients reported considerable rates of both pain frequency and intensity. Quartile (Q) splits of the *visual analogue pain frequency* scale revealed *n* = 486 patients (39.3%) as falling into Q1, 198 (16.0%) into Q2, 163 (13.2%) in Q3 and 377 (30.5%) in Q4. For the *visual analogue pain intensity scale*, *n* = 667 patients (53.9%) fell into Q1, 282 (22.8%) into Q2, 192 (15.5%) in Q3 and 83 (6.7%) in Q4). All constructs were significantly interrelated (**Hypothesis 1**). Correlation coefficients are given in the Online Supplemental Material (S1 Table).

**Table 2:** Means, standard deviations and symptom level descriptors for the total sample (*N* = 1238 patients with chronic tinnitus).

	*M*	*SD*	*Symptom level descriptors (M +/- 1 SD)*
**Tinnitus-related distress [Tinnitus Questionnaire–German version, TQ]**			
Total	39.55	17.07	[n/a]
**Pain characteristics [Visual Analogue Scales]**			
Frequency	4.56	3.66	[n/a]
Intensity	2.80	2.59	[n/a]
**Pain perception [Pain Perception Scale, SES]**			
Affective	24.23	10.08	[average*]
Sensory	13.75	5.12	[average*]
**Psychological comorbidities [ICD-10 Symptom Rating, ISR]**			
Total	0.81	0.59	[mild–moderate**]
Depressive syndrome	1.18	0.92	[mild–moderate**]
Anxiety-related syndrome	0.93	0.91	[elevated–mild**]
Obsessive-compulsive syndrome	0.78	0.87	[elevated–mild**]
Somatoform syndrome	0.62	0.81	[elevated–moderate**]
Eating-related syndrome	0.68	0.81	[mild–moderate**]
Supplementary scale	0.75	0. 57	[n/a]
**Depressivity [Center for Epidemiological Studies Depression Scale, ADS]**			
Total	18.33	11.85	[n/a]
**Perceived stress [Perceived Stress Questionnaire, PSQ]**			
Total	0.46	0.18	[normal–mildly elevated**]
Tension	0.59	0.22	[normal–moderately elevated**]
Worries	0.40	0.23	[normal–mildly elevated**]
Joy	0.48	0.22	[normal–mildly depleted**]
Demands	0.50	0.23	[normal–mildly elevated**]
**Coping attitudes [Self-Efficacy-Optimism-Pessimism-Scale, SWOP]**			
Self-efficacy	2.76	0.58	[n/a]
Optimism	2.72	0.76	[n/a]
Pessimism	2.14	0.72	[n/a]

*M* = mean, *SD* = standard deviation; n/a = not applicable. Degrees of symptom levels: PSQ: normal, mildly elevated / depleted, moderately elevated, severely elevated; ISR: normal, elevated, mild, moderate, severe.

* relative to a clinical sample of patients with pain perceptions

** relative to the general population

### Mediation analyses

#### Overview

The associations between tinnitus-related distress and pain perceptions were mediated by most psychological comorbidities and process variables. Depressivity emerged as a key factor in mediating the effects of psychological comorbidities on affective and sensory pain perceptions. Anxiety-based comorbidities appear to influence [a] tinnitus-related distress and affective pain perceptions through heightened perceived stress and reduced coping abilities and [b] tinnitus-related distress and sensory pain perceptions through heightened worry and pessimism. Somatization-based comorbidities appear to additionally influence sensory pain perceptions through heightened emotional tension. The following paragraphs yield a more detailed description of results.

*Simple and parallel multiple mediator analyses (independent variable: TQ; dependent variables: SES_A or SES_S; mediating variables: ISR, ADS, PSQ, SWOP)*.

#### Hypothesis 2

**Fig 2:**
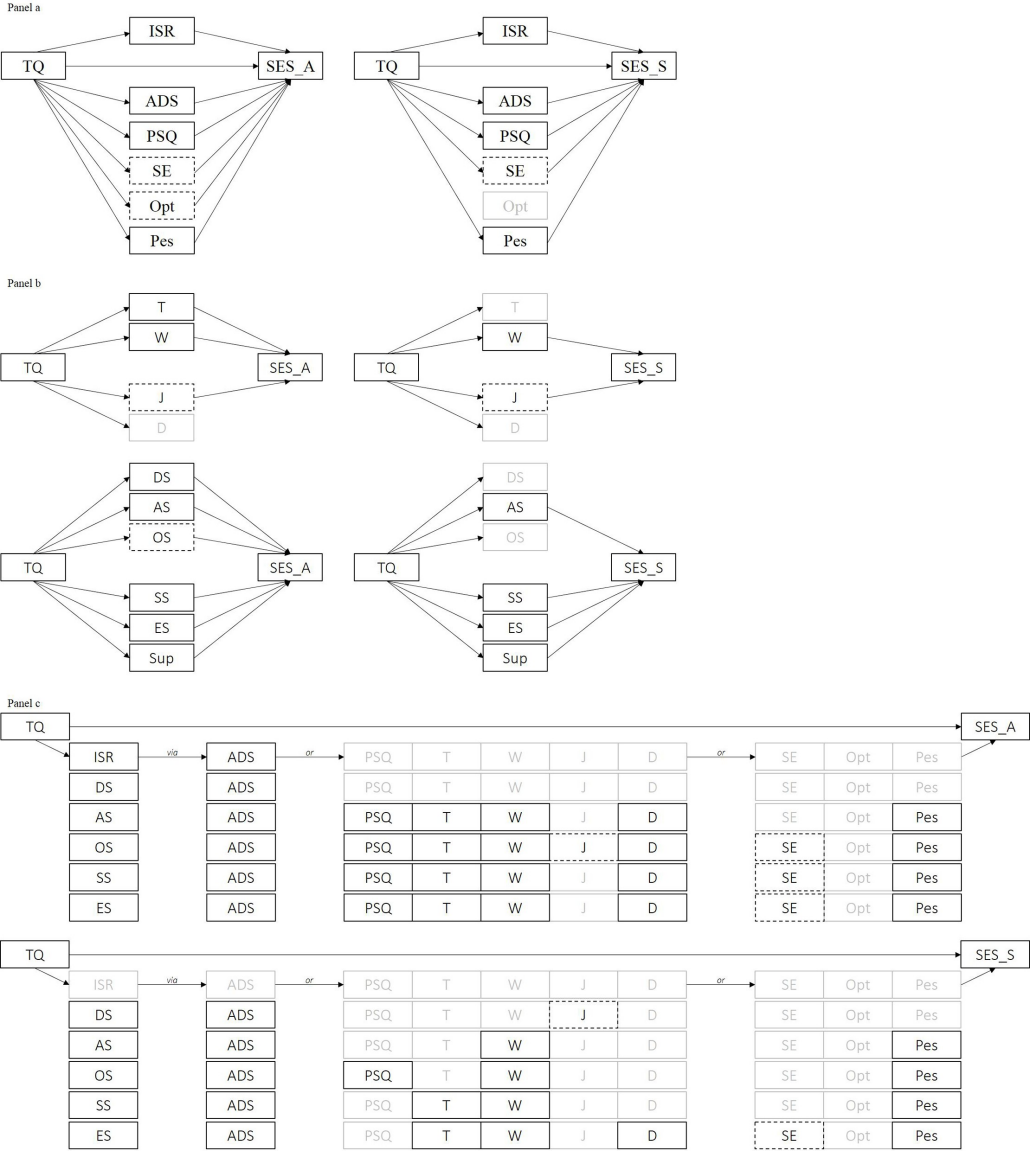
Graphical illustration of significant indirect effects. Black box frames indicate significant positive, dotted box frames significant negative and greyed-out boxes non-significant indirect effects. *Panel a*: Results of the simple mediator models for affective (left) or sensory pain perception (right). *Panel b*: Results of the parallel multiple mediator models for PSQ- (upper row) and ISR subscales (lower row) mediating affective (left) or sensory pain perception (right). *Panel c*: Results of the serial multiple mediator models that examine the effects of shared psychological process variables across psychological ‘comorbidities” on affective (upper row) or sensory pain perception (lower row). *Reading example for upper row*: *the indirect effect of tinnitus-related distress [TQ] on affective pain perception [SES_A] through psychological comorbidities [ISR] is explained by the latters impact on depressivity [ADS]*, *but not perceived stress [PSQ*, *T*, *W*, *reduced J*, *D] or coping attitudes [reduced SE*, *Opt*, *heightened Pes]*. TQ = Tinnitus Questionnaire–German version total score, SES_A = Affective Pain Perception Scale: SES_S = Sensory Pain Perception Scale, ISR = ICD-10 Symptom Rating total score, DS = depressive syndrome, AS = anxiety-related syndrome, OS = obsessive-compulsive syndrome, SS = somatoform syndrome, ES = eating-related syndrome, Sup = supplementary scale, ADS = Center for Epidemiological Studies Depression Scale total score, PSQ = Perceived Stress Questionnaire total score, T = tension, W = worries, J = joy, D = demands, SE = Self-efficacy scale, Opt = Optimism scale; Pes = Pessimism scale. Significance level set at *p* < .05.

Simple mediation analyses revealed significant indirect effects of tinnitus-related distress on affective and sensory pain perceptions via the total scores of most measured mediating variables. Optimism mediated the relationship between tinnitus-related distress and affective, but not sensory pain perception (Fig 2, *Panel a*). Parallel multiple mediator analyses of the PSQ subscales revealed significant indirect effects of worry and lack of joy on both types of pain perceptions, with tension exerting an influence on affective pain perception only. Demands did not exert an indirect effect on either pain perception index. Analysis of the ISR subscales revealed that all psychological comorbidities mediated the relationship between tinnitus-related distress and affective pain perception. By contrast, the relationship between tinnitus-related distress and sensory pain perception was mediated by anxiety-, somatoform, and eating-related symptoms only (*Panel b*).

*Serial multiple mediator analyses (independent variable: TQ; first-level mediating variables: ISR subscale scores; second-level mediating variables: ADS, PSQ, SWOP scores; dependent variables: SES_A or SES_S)*.

#### Hypothesis 3

Serial multiple mediator analyses revealed that depressivity partly explained the indirect effects of psychological comorbidities on the relationship between tinnitus-related distress and *affective pain perception*. A more anxiety-based comorbidity cluster (anxiety-, obsessive-compulsive, somatoform and eating-related syndromes) further appeared to exert influence through its impact on perceived stress, self-efficacy and pessimism. Indirect effects that explained the relationship between tinnitus-related distress and *sensory pain perception* were also mediated by depressivity. Here, however, anxiety-based comorbidities affected sensory pain primarily through heightened worry and pessimism. Somatoform and eating-related syndromes were further associated with heightened emotional tension (Fig 2, *Panel c*). All path coefficients are provided in the Online Supplemental Material (S2 Table).

### Tinnitus decompensation

In the current sample, *n* = 810 patients (65.4%) reported compensated and 428 patients (34.6%) decompensated tinnitus-related distress levels. For *affective pain perceptions*, *n* = 640 (51.7%) reported below-, 533 (43.1%) average and 65 (5.3%) above average levels. For *sensory pain perceptions*, *n* = 569 (46.0%) reported below-, 618 (49.9%) average and 51 (4.1%) above average levels relative to the pain perceptions reference sample. Table 3, *Panel a* provides a categorical tinnitus- x pain-related distress frequency matrix. Means, standard deviations and group comparisons for patients with decompensated vs. compensated tinnitus are reported in Table 3, *Panel b*.

**Table 3:** Panel a: Frequencies across tinnitus- x pain-related distress categories. Panel b: Means, standard deviations, comparisons of means and effect sizes d for the total, compensated and decompensated patient samples.

**A**						
**Tinnitus-related distress**	**Affective pain perception**					
	Below average (n)	Average (n)	Above average (n)			
Compensated	529 (82.7%)	273 (51.2%)	8 (12.3%)			
Decompensated	111 (17.3%)	260 (48.8%)	57 (87.7%)			
	**Sensory pain perception**					
Compensated	453 (79.6%)	347 (56.1%)	10 (19.6%)			
Decompensated	116 (20.4%)	271 (43.9%)	41 (80.4%)			
**B**						
Subsample	Compensated (*n* = 810)		Decompensated (*n* = 428)			
	*M*	*SD*	*M*	*SD*	Group effect	*d (CI)*
**Pain characteristics [Visual Analogue Scales]**						
Frequency	3.95	3.55	5.73	3.58	*F*(1, 1223) = 68.96***	0.50 (0.38–*0*.*62*)
Intensity	2.12	2.16	4.1	2.83	*F*(1, 1223) = 186.02***	*0*.*79* (*0*.*76*–**1.00**)
**Pain perception [Pain Perception Scale, SES]**						
Affective	20.7	7.1	30.91	11.43	*F*(1, 1237) = 373.44***	**1.16** (**1.03**–**1.28**)
Sensory	12.34	3.44	16.42	6.35	*F*(1, 1237) = 207.57***	**0.88** (*0*.*75*–**1.00**)
**Depressivity [Center for Epidemiological Studies Depression Scale, ADS]**						
Total	13.82	9.5	26.96	11.08	*F*(1, 1158) = 444.92***	**1.31** (**1.17**–**1.44**)
**Perceived stress [Perceived Stress Questionnaire, PSQ]**						
Total	0.41	0.16	0.56	0.16	*F*(1, 1235) = 246.16***	**0.94** (**0.81**–**1.06**)
Tension	0.52	0.21	0.72	0.19	*F*(1, 1235) = 280.31***	**0.98 (0.86**–**1.11**)
Worries	0.33	0.2	0.53	0.21	*F*(1, 1235) = 251.50***	**0.98** (**0.86**–**1.11**)
Joy	0.53	0.21	0.37	0.19	*F*(1, 1235) = 168.20***	*-0*.*79* (-*0*.*67*- -**0.91**)
Demands	0.47	0.22	0.54	0.24	*F*(1, 1235) = 26.69***	0.31 (0.19–0.43)
**Psychological comorbidities [ICD-10 Symptom Rating, ISR]**						
Total	0.63	0.47	1.17	0.64	*F*(1, 1183) = 271.73***	**1.01** (**0.89**–**1.14**)
Depressive syndrome	0.87	0.76	1.78	0.91	*F*(1, 1183) = 327.77***	**1.12** (**1.00–1.25**)
Anxiety-related syndrome	0.72	0.74	1.36	1.05	*F*(1, 1183) = 144.69***	*0*.*75* (*0*.*62*–**0.87**)
Obsessive-compulsive syndrome	0.63	0.77	1.1	0.96	*F*(1, 1183) = 83.30***	*0*.*56* (0.44–*0*.*68*)
Somatoform syndrome	0.44	0.67	0.97	0.93	*F*(1, 1183) = 122.19***	*0*.*69* (*0*.*57*–**0.81**)
Eating-related syndrome	0.44	0.67	0.97	0.93	*F*(1, 1183) = 122.19***	*0*.*69* (*0*.*57*–**0.81**)
Supplementary scale	0.62	0.74	0.79	0.92	*F*(1, 1183) = 11.74**	0.21 (0.10–0.33)
**Coping attitudes [Self-Efficacy-Optimism-Pessimism-Scale, SWOP]**						
Self-efficacy	2.89	0.53	2.52	0.6	*F*(1, 1237) = 124.72***	*-0*.*67* (-*0*.*55*- -0.79)
Optimism	2.88	0.69	2.41	0.79	*F*(1, 1237) = 116.75***	*-0*.*65* (-*0*.*56*- -**0.80**)
Pessimism	1.98	0.66	2.45	0.73	*F*(1, 1237) = 134.82***	*0*.*69* (*0*.*59*–**0.84**)

Within affective or sensory pain perception indices, all horizontal and vertical cell comparisons significantly differ from each other (*chi*^*2*^ *p* <. 05_Bonferroni corrected_). Percentages are referring to respective pain perception categories.

M = mean, SD = standard deviation, d = Cohen’s d (small effect d >.20 < .50; *medium effect* > .50 < .80; large effect > .80). CI = 95% Confidence Interval

#### Hypothesis 4

Compared to patients with compensated tinnitus, patients with decompensated tinnitus reported significantly higher symptom burden across all measured indices with pain perceptions, depressivity, perceived stress (tension, worries) as well as psychological comorbidities (depressive syndrome) yielding large, and [reduced] joy, remaining psychological comorbidities and coping attitudes yielding medium-effect-size differences.

*Moderation analyses investigating the impact of tinnitus decompensation vs. compensation on relations between tinnitus-related distress, mediating variables, and affective or sensory pain perception*.

#### Hypothesis 5

**Fig 3:**
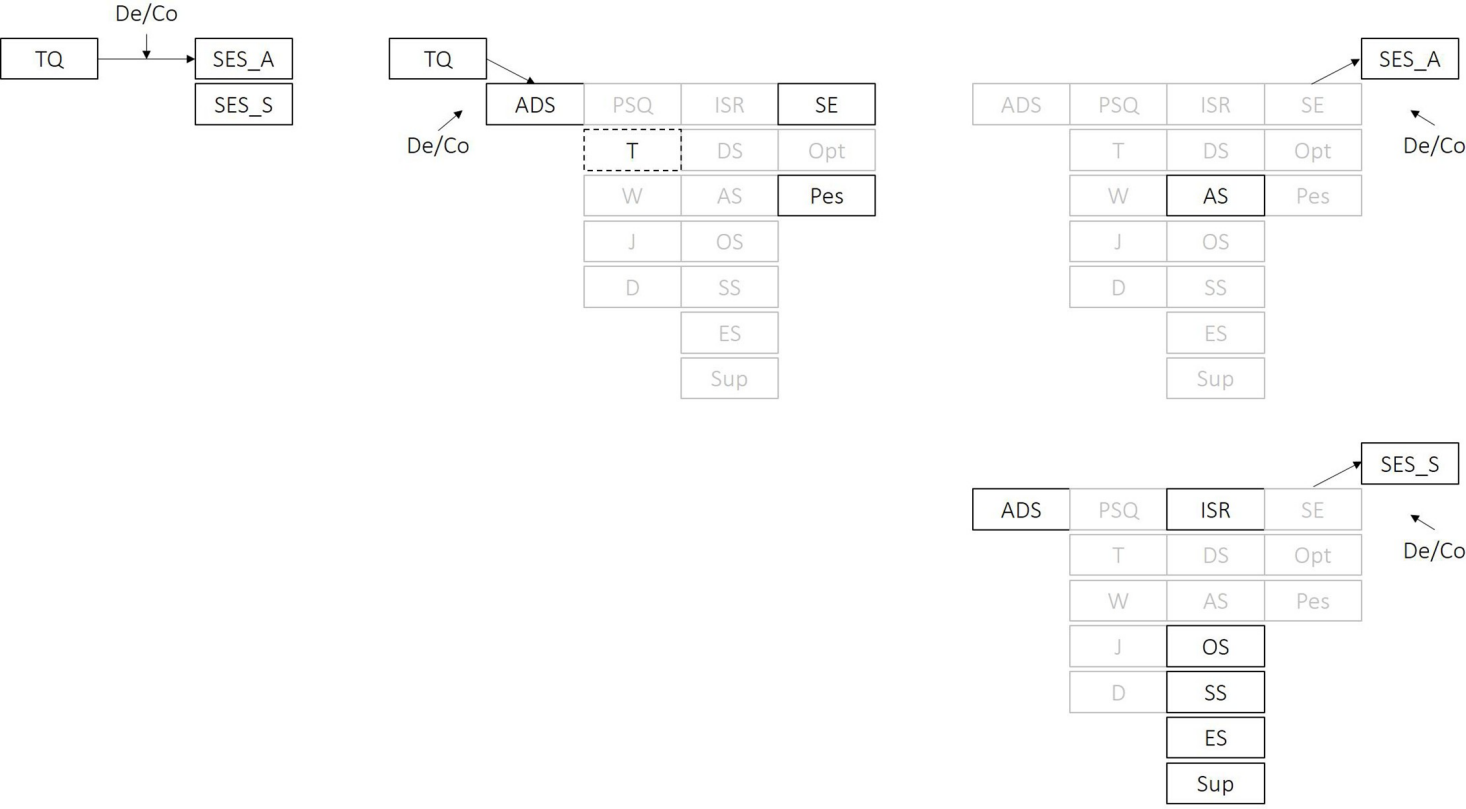
Graphical illustration of simple moderation effects. *De/Co* indicates the specification of tinnitus decompensation vs. compensation as a putative moderator of paths *c* (left), *a* (middle) and *b* (right upper row: affective pain perception; lower row: sensory pain perception). Continuous black box frames indicate that respective effects are stronger in patients with decompensated vs. compensated tinnitus, dotted box frames the opposite. Greyed out boxes indicate non-moderated effects. TQ = Tinnitus Questionnaire–German version total score, SES_A = Affective Pain Perception Scale: SES_S = Sensory Pain Perception Scale, ADS = Center for Epidemiological Studies Depression Scale total score, PSQ = Perceived Stress Questionnaire total score, T = tension, W = worries, J = joy, D = demands, ISR = ICD-10 Symptom Rating total score, DS = depressive syndrome, AS = anxiety-related syndrome, OS = obsessive-compulsive syndrome, SS = somatoform syndrome, ES = eating-related syndrome, Sup = supplementary scale, SE = Self-efficacy scale, Opt = Optimism scale; Pes = Pessimism scale. Significance levels were set at *p* < .05.

Moderation analyses for each pathway revealed significant differences for patients with decompensated vs. compensated tinnitus in the extents to which tinnitus-related distress was related to affective and sensory pain perception indices as well as depressivity, emotional tension, self-efficacy and pessimism. Tinnitus decompensation further appeared to exacerbate relationships between [1] anxiety and affective pain perception, and [2] depressive, obsessive-compulsive, somatoform, and eating-related difficulties and sensory pain. Tinnitus decompensation did not significantly impact upon relationships between PSQ and SWOP indices on affective or sensory pain perception respectively. See Fig 3 for a graphical illustration of significant differences in path coefficients for patients with decompensated vs. compensated tinnitus (S3 Table**)**.

### Moderated mediation analyses

#### Hypothesis 6

**Fig 4:**
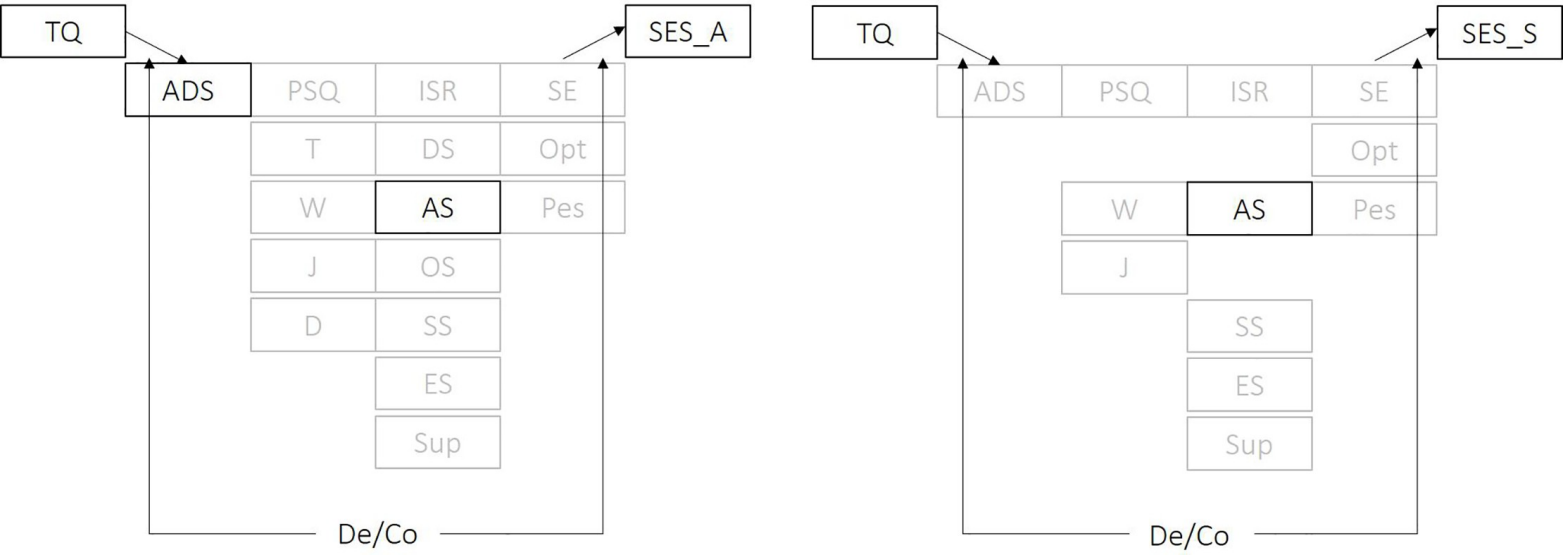
Graphical illustration of moderated mediation models for affective (left) and sensory pain perception (right). *De/Co* indicates the specification of tinnitus decompensation vs. compensation as a putative moderator of the indirect effects of tinnitus-related distress on affective or sensory pain perceptions through the specified process variables. Continuous black box frames indicate that respective indirect effects are stronger in patients with decompensated vs. compensated tinnitus. Greyed out boxes indicate non-moderated indirect effects. TQ = Tinnitus Questionnaire–German version total score, SES_A = Affective Pain Perception Scale: SES_S = Sensory Pain Perception Scale, ADS = Center for Epidemiological Studies Depression Scale total score, PSQ = Perceived Stress Questionnaire total score, T = tension, W = worries, J = joy, ISR = ICD-10 Symptom Rating total score, DS = depressive syndrome, AS = anxiety-related syndrome, OS = obsessive-compulsive syndrome, SS = somatoform syndrome, ES = eating-related syndrome, Sup = supplementary scale, SE = Self-efficacy scale, Opt = Optimism scale; Pes = Pessimism scale. Significance level set at *p* < .05.

Last, moderated mediation analyses revealed significantly stronger indirect effects of tinnitus-related distress through [1] anxiety (ISR) on both pain perception indices and [2] depressivity (ADS) on affective, but not sensory pain perception in patients with decompensated vs. compensated tinnitus (Fig 4). All coefficients are provided in the Online Supplemental Material (S4 Table).

## Discussion

Variations in depressivity, internal stress reactions, psychological comorbidities and coping attitudes underlay observed relationships between tinnitus-related distress and affective and sensory pain perceptions in a sample of patients with chronic tinnitus. Thus, conceptualizing and therapeutically addressing individual interactions of these psychological constructs may contemporaneously attenuate both symptom clusters. These findings are in keeping with findings that have repeatedly highlighted the effectiveness of psychological treatments for relieving tinnitus- [13, 67, 68] and pain-related distress [69, 70, 71].

Optimism, tension [PSQ], depressivity [ADS], and obsessive-compulsive symptoms [ISR] mediated the link between tinnitus-related distress and affective, but not sensory pain perception. Viewed from an emotion regulation perspective, the observed negative indirect effect of obsessions (with higher obsessive symptoms reducing affective pain perception) might point to a possible function of these symptoms in regulating underlying affective states [72,73,74,75]. Similarly, emotional tension has been described in individuals with difficulties in identifying or regulating emotions [76]–which have also been observed in individuals with psychosomatic symptoms or emotionally avoidant coping styles [77,78,79,80]. The positive impact of optimism on tinnitus-related distress or pain perception has been demonstrated before [81, 20, 82], and increasing optimism thus constitutes a target for psychological interventions [83, 84]. Pessimism, conversely, has been associated with heightened inducibility of pain perceptions in healthy individuals [85], and therefore also warrants psychological targeting within broader therapeutic strategies aimed to reduce depressivity [86]. Overall, coping attitudes may constitute important vulnerability or maintaining factors for the co-occurrence of tinnitus-related distress and pain perceptions.

Investigating whether psychological comorbidities accounted for the co-occurrence of tinnitus-related distress and pain perceptions *through* affecting depressivity, perceived stress or coping attitudes, depressivity emerged as a key factor that determined the impact of all psychological comorbidities. Comorbidities that may contain aspects of affective avoidance such as anxiety, obsessive-compulsive, somatoform and eating-related syndromes [87,88] were found to affect the link between tinnitus-related distress and *affective pain perception* by exacerbating perceived stress and pessimism alongside lowering self-efficacy beliefs. For *sensory pain perception*, a broadly similar pattern was observed; here, however, worry and pessimism influenced sensory pain perceptions more strongly. This finding is in keeping with studies highlighting the role of these factors in influencing anxiety in the context of sensory misperceptions [89, 90, 91, 92]. Emotional tension—which was primarily associated with affective pain perceptions—may similarly be addressed by applied emotion regulation interventions [93, 94, 95].

Tinnitus decompensation was associated with [1] considerably higher symptom burden across all measured psychological indices, [2] stronger relationships between tinnitus-related distress and [a] both types of pain perception (paths *c*) as well as [b] depressivity and reduced coping attitudes (paths *a*), [3] stronger relationships between anxiety and *affective*, and depressivity, obsessive-compulsive, somatoform, as well as eating-related symptoms and *sensory* pain perception (paths *b*). Tinnitus decompensation was further associated with [4] significantly stronger indirect effects of depressivity and anxiety in mediating affective pain perceptions; and anxiety in mediating sensory pain perceptions.

Tinnitus decompensation has previously been associated with heightened psychological distress across different domains of experience [96, 97, 45, 98]. These, as well as the current findings may reflect conceptual similarities between tinnitus-related distress and related psychological constructs–such as pain perceptions, depressivity, pessimism, and reduced self-efficacy beliefs. The exacerbation of relations between the specified mediators and sensory, but not affective pain perceptions with tinnitus decompensation may potentially point to a somatoform shift in emotional experience with higher emotional distress being associated with a stronger sensory focus on psychophysiological experience. If this were the case, future studies should investigate reversed u-shaped relationships between sensory perceptions and patients’ emotional experiences. Interestingly, the relationship between tinnitus-related distress and emotional tension *decreased* alongside increasing severity of these factors. It may also be speculated that increasing tinnitus-related distress might lead to a chronification process that involves perceived stress becoming an independent risk factor [99, 100, 101]. Alternatively, perceived emotional tension might shift towards tinnitus-related distress in an effort to regulate emotional destabilization.

In keeping with previous studies that highlight the roles of depression and anxiety in patients with chronic tinnitus [102, 103] or pain [104, 105, 106], tinnitus decompensation appeared to exacerbate the impact of anxiety on affective, and depressivity and anxiety-centred symptoms on sensory pain perceptions. In addition to the psychological impact of these symptom clusters, physiological arousal may also influence fear of pain thereby forming a possible link between anxiety and pain perceptions [107]. Similarly, the impact of depressivity on altered sensory perceptions has also been highlighted [108]. The moderated mediation analyses’ findings thus highlight that depressivity and anxiety take center stage in underlying the co-occurrence of tinnitus-related distress and pain perceptions.

### Strength and limitations

The current study has several limitations. Most importantly, the cross-sectional nature of the data as well as the absence of a control group limit its causal interpretability and generalizability. Cross-sectional mediation analyses do not imply causation; however, they are suited to generate causal hypotheses that ought to be tested in future prospective or experimental studies. Partial mediation of the observed associations further suggests the existence of important third variables that need to be theoretically deduced, measured and interactionally examined in future studies. Whilst the study conceptualized patients’ tinnitus and pain perceptions within a broader biopsychosocial framework [28, 109] it did not stratify patients’ pain ratings according to the presence or absence of specific medical conditions [110] thereby limiting the identification of differentially caused sensory pain stimuli. Similarly, information about pain or antidepressant medication was not available. The visual analogue scales that we used to quantify pain frequency and intensity have been subject of scientific controversy [111, 112]; however were chosen for reasons of clinical feasibility. Last, given the exploratory nature of the study, we used lenient tests for possible indirect effects. We preferred committing Type I over Type II errors at this stage of empirical investigation into identifying common psychological pathways between tinnitus-related distress and pain perceptions. Consequently, however, the findings need to be cautiously interpreted and replicated in future studies.

Notwithstanding, the present study is the first to investigate the co-occurrence of chronic tinnitus and pain perceptions in a large clinical sample of patients with chronic tinnitus. It provides important first insights into the roles of psychological factors that explain shared variance between the two symptom clusters thus highlighting their importance in conceptualizing and treating these syndromes. Whilst depressivity emerged as a key factor, associated constructs such as perceived stress (in particular worry and emotional tension) and subjectively impaired coping attitudes constitute promising intervention targets. Locating the pathways through which psychological processes may generate distress gives way to conceptualizing and testing transdiagnostic psychological treatment approaches that improve the well-being of patients with chronic tinnitus with or without concurrent pain symptoms. Whilst some studies conceptualize psychological distress within a diagnostic framework that assumes the presence of “comorbidities” as distinct clinical entities that exist in addition to an “index disease” such as chronic tinnitus or -pain [113,114], the present study challenges the helpfulness of this view. It seems somewhat ill-suited to conceptualize and treat separate conditions suited given the co-occurring and functionally similar psychosocial conditions that patients with chronic tinnitus commonly face [115, 116, 59].

## Conclusions

Results of the present study point to a key role of *psychological processes* as common denominators that may account for co-occurrences of chronic tinnitus, pain perceptions and psychological “comorbidities”. Transdiagnostic interventions that focus on shared cognitive-emotional factors are thereby likely to reduce the distress associated with co-occurring syndromatic conditions [117, 118]. Indeed, such treatment approaches have been gaining momentum in offering useful tools to conceptualize and treat co-occurring symptom clusters [119, 115, 120, 121]. Any such intervention may prevent symptom chronification or alleviate distress by developing individualised case conceptualizations and thereon based idiosyncratic treatment plans that may feature a range of interventions aimed at modifying individual interactions of memories, situational stimulus interpretations, habitual or current emotional states and behaviours. Future research needs to continue to investigate interactions of psychological process variables pertinent to tinnitus-related distress and co-occurring affective or sensory phenomena.

## Supporting information

S1 Table Correlation matrix for the obtained measures. (DOCX)

S2 Table Path coefficients for significant indirect effects. (DOCX)

S3 Table Simple moderation effects for paths *c*, *a* and *b*. (DOCX)

S4 Table Moderated mediation effects for affective and sensory pain perception. (DOCX)
