# Supplementary material for: Tinnitus-related distress and pain perceptions in patients with chronic tinnitus – Do psychological factors constitute a link?
Source: PLoS One. 2020 Jun 25;15(6):e0234807. doi: 10.1371/journal.pone.0234807 (PMC7316290; doi:10.1371/journal.pone.0234807)
Supplement: S1 Table — (DOCX) [file pone.0234807.s001.docx]

**Supplementary data - Table 1**: Correlation matrix for the obtained measures.

|  |  | SES_A | SES_S | ADS | PSQ |  |  |  |  | ISR |  |  |  |  |  |  | SE | Opt | Pes |
| --- | --- | --- | --- | --- | --- | --- | --- | --- | --- | --- | --- | --- | --- | --- | --- | --- | --- | --- | --- |
|  |  |  |  |  |  | T | W | J* | D |  | DS | AS | OS | SS | ES | Sup |  |  |  |
| TQ | | .55 | .43 | .63 | .51 | .52 | .50 | -.44 | .21 | .50 | .55 | .40 | .28 | .33 | .13 | .51 | -.40 | -.39 | -36 |
| SES_A | |  | .76 | .53 | .42 | .41 | .42 | -.32 | .20 | .51 | .49 | .41 | .29 | .38 | .20 | .49 | -.32 | -.30 | .30 |
| SES_S | |  |  | .41 | .33 | .29 | .33 | -.22 | .16 | .44 | .37 | .37 | .25 | .34 | .19 | .42 | -.25 | -.20 | .23 |
| ADS | |  |  |  | .72 | .68 | .73 | -.66 | .33 | .70 | .80 | .55 | .46 | .41 | .16 | .69 | -.58 | -.60 | .42 |
| PSQ | |  |  |  |  | .85 | .87 | -.79 | .74 | .62 | .71 | .48 | .43 | .37 | .16 | .58 | -.55 | -.55 | .41 |
|  | T |  |  |  |  |  | .67 | -.68 | .54 | .56 | .66 | .43 | .39 | .30 | .12 | .55 | -.48 | -.49 | .33 |
|  | W |  |  |  |  |  |  | -.67 | .50 | .63 | .71 | .50 | .43 | .39 | .17 | .59 | -.55 | -.57 | .45 |
|  | J* |  |  |  |  |  |  |  | -.38 | -.52 | -.64 | -.38 | -.36 | -.31 | -.09^Ɨ^ | -.49 | .59 | .59 | -.36 |
|  | D |  |  |  |  |  |  |  |  | .29 | .33 | .21 | .21 | .18 | .11 | .25 | -.22 | -.19 | .17 |
| ISR | |  |  |  |  |  |  |  |  |  | .82 | .80 | .77 | .71 | .50 | .88 | -.51 | -.48 | .35 |
|  | DS |  |  |  |  |  |  |  |  |  |  | .62 | .56 | .47 | .21 | .77 | -.55 | -.56 | .39 |
|  | AS |  |  |  |  |  |  |  |  |  |  |  | .57 | .51 | ,25 | .63 | -.46 | -.38 | .25 |
|  | OS |  |  |  |  |  |  |  |  |  |  |  |  | .45 | .28 | .60 | -.36 | -.37 | .21 |
|  | SS |  |  |  |  |  |  |  |  |  |  |  |  |  | .27 | .54 | -.30 | -.28 | .23 |
|  | ES |  |  |  |  |  |  |  |  |  |  |  |  |  |  | .31 | -.12 | -.10 ^Ɨ^ | .11 |
|  | Sup |  |  |  |  |  |  |  |  |  |  |  |  |  |  |  | -.47 | -.45 | .35 |
| SE | |  |  |  |  |  |  |  |  |  |  |  |  |  |  |  |  | .65 | -.35 |
| Opt | |  |  |  |  |  |  |  |  |  |  |  |  |  |  |  |  |  | -.36 |
| *Notes*: TQ = Tinnitus Questionnaire – German version total score, SES_A = Affective Pain Perception Scale: SES_S = Sensory Pain Perception Scale, ISR = ICD-10 Symptom Rating total score, DS = depressive syndrome, AS = anxiety-related syndrome, OS = obsessive-compulsive syndrome, SS = somatoform syndrome, ES = eating-related syndrome, Sup = supplementary scale, ADS = Center for Epidemiological Studies Depression Scale total score, PSQ = Perceived Stress Questionnaire total score, T = tension, W = worries, J = joy [*reversely coded – lower scores indicating higher distress], D = demands, SE = Self-efficacy Scale, Opt = Optimism Scale; Pes = Pessimism Scale. All correlation coefficients *p* < .001 (except *Ɨ = p* < . 01). | | | | | | | | | | | | | | | | | | | |
