# Supplementary material for: Tinnitus-related distress and pain perceptions in patients with chronic tinnitus – Do psychological factors constitute a link?
Source: PLoS One. 2020 Jun 25;15(6):e0234807. doi: 10.1371/journal.pone.0234807 (PMC7316290; doi:10.1371/journal.pone.0234807)
Supplement: S2 Table — (DOCX) [file pone.0234807.s002.docx]

**Supplementary data - Table 2.** Path coefficients for significant indirect effects.

*Panel a: Path coefficients and confidence intervals for the simple mediator models (independent variable: TQ; dependent variables: SES_A or SES_S; mediating variables: ISR, ADS, PSQ, SE, Opt, and Pes)*

|  | *a* | *se* | *LL*  *CI* | *UL*  *CI* | *b* | *se* | *LL*  *CI* | *UL*  *CI* | *c* | *se* | *LL*  *CI* | *UL*  *CI* | *c‘* | *se* | *LL*  *CI* | *UL*  *CI* | *ab* | *se* | *LL*  *CI* | *UL*  *CI* |
| --- | --- | --- | --- | --- | --- | --- | --- | --- | --- | --- | --- | --- | --- | --- | --- | --- | --- | --- | --- | --- |
| **TQ-SES_A** |  |  |  |  |  |  |  |  |  |  |  |  |  |  |  |  |  |  |  |  |
| **ADS** | .44 | .02 | .41 | .47 | .24 | .03 | .19 | .29 | .34 | .01 | .31 | .36 | .23 | .02 | .19 | .26 | .11 | .01 | .08 | .13 |
| **PSQ** | .01 | .00 | .00 | .01 | 10.76 | 1.53 | 7.76 | 13.76 | .33 | .01 | .30 | .35 | .27 | .02 | .24 | .30 | .06 | .01 | .04 | .08 |
| **ISR** | .02 | .00 | .02 | .02 | 5.31 | .45 | 4.42 | 6.19 | .33 | .01 | .30 | .36 | .24 | .02 | .21 | .27 | .10 | .01 | .07 | .12 |
| **SE** | -.01 | .00 | -.02 | -.01 | -2.01 | .45 | -2.88 | -1.14 | .33 | .01 | .30 | .35 | .30 | .02 | .27 | .33 | .03 | .01 | .01 | .04 |
| **Opt** | -.02 | .00 | -.02 | -.02 | -1.29 | .34 | -1.96 | -.62 | .33 | .01 | .30 | .35 | .30 | .02 | .27 | .33 | .02 | .01 | .01 | .04 |
| **Pes** | .02 | .00 | .01 | .02 | 1.56 | .35 | .87 | 2.25 | .33 | .01 | .30 | .35 | .30 | .02 | .27 | .33 | .02 | .01 | .01 | .04 |
| **TQ-SES_S** |  |  |  |  |  |  |  |  |  |  |  |  |  |  |  |  |  |  |  |  |
| **ADS** | .44 | .02 | .41 | .47 | .10 | .02 | .10 | .12 | .13 | .01 | .12 | .15 | .09 | .01 | .07 | .11 | .04 | .01 | .03 | .06 |
| **PSQ** | .01 | .00 | .01 | .01 | 4.17 | .85 | 2.50 | 5.84 | .13 | .01 | .11 | .14 | .11 | .01 | .09 | .12 | .02 | .01 | .01 | .03 |
| **ISR** | .02 | .00 | .02 | .02 | 2.56 | .25 | 2.06 | 3.05 | .13 | .01 | .11 | .14 | .08 | .01 | .07 | .10 | .04 | .01 | .03 | .06 |
| **SE** | -.01 | .00 | -.02 | -.01 | -.83 | .25 | -1.31 | -.35 | .13 | .01 | .11 | .14 | .12 | .01 | .10 | .13 | .01 | .00 | .00 | .02 |
| **Pes** | .02 | .00 | .01 | .02 | .59 | .20 | .21 | .97 | .13 | .01 | .11 | .14 | .12 | .01 | .10 | .14 | .01 | .00 | .00 | .02 |

*Panel b: Path coefficients and confidence intervals for the parallel multiple mediator models (independent variable: TQ; dependent variables: SES_A or SES_S; mediating variables: PSQ and ISR subscales).*

|  | *a* | *se* | *LL*  *CI* | *UL*  *CI* | *b* | *se* | *LL*  *CI* | *UL*  *CI* | *ab* | *se* | *LL*  *CI* | *UL*  *CI* | *p_ab_* |
| --- | --- | --- | --- | --- | --- | --- | --- | --- | --- | --- | --- | --- | --- |
| **TQ-SES_A** |  |  |  |  |  |  |  |  |  |  |  |  |  |
| **PSQ T** | .01 | .00 | .01 | .01 | 5.73 | 1.70 | 2.93 | 8.53 | .04 | .01 | .02 | .06 | .00 |
| **W** | .01 | .00 | .01 | .01 | 7.58 | 1.59 | 4.86 | 10.09 | .05 | .01 | .03 | .07 | .00 |
| **J** | -.01 | .00 | -.01 | -.01 | 2.82 | 1.59 | .21 | 5.43 | -.02 | .01 | -.03 | -.00 | .08 |
| **ISR DS** | .03 | .00 | .03 | .03 | 1.14 | .43 | .43 | 1.85 | .03 | .01 | .01 | .06 | .01 |
| **AS** | .02 | .00 | .02 | .02 | .94 | .36 | .35 | 1.53 | .02 | .01 | .01 | .03 | .01 |
| **OS** | .01 | .00 | .01 | .02 | -.75 | .36 | -1.33 | -.16 | -.01 | .01 | -.02 | -.00 | .04 |
| **SS** | .02 | .00 | .01 | .02 | 1.27 | .36 | .69 | 1.85 | .02 | .01 | .01 | .03 | .00 |
| **ES** | .01 | .00 | .00 | .01 | .68 | .30 | .19 | 1.18 | .00 | .00 | .00 | .01 | .05 |
| **Sup** | .02 | .00 | .02 | .02 | 2.43 | .72 | 1.24 | 3.62 | .04 | .02 | .02 | .07 | .00 |
| **TQ-SES_S** |  |  |  |  |  |  |  |  |  |  |  |  |  |
| **PSQ W** | .01 | .00 | .01 | .01 | 4.23 | .88 | 2.77 | 5.68 | .09 | .01 | .02 | .04 | .00 |
| **J** | -.01 | .00 | -.01 | -.01 | 2.34 | .88 | .89 | 3.79 | -.01 | .01 | -.02 | -.01 | .01 |
| **ISR AS** | .02 | .00 | .02 | .02 | .75 | .20 | .42 | 1.08 | .02 | .00 | .01 | .02 | .00 |
| **SS** | .02 | .00 | .01 | .02 | .67 | .20 | .35 | 1.00 | .01 | .00 | .01 | .02 | .00 |
| **ES** | .01 | .00 | .00 | .01 | .38 | .17 | .10 | .65 | .00 | .00 | .00 | .01 | .05 |
| **Sup** | .02 | .00 | .02 | .02 | 1.68 | .40 | 1.02 | 2.34 | .03 | .01 | .01 | .04 | .00 |

*Panel c: Serial mediator models for affective or sensory pain perception (independent variable: TQ; first-level mediating variables: ISR subscale scores; second-level mediating variables: ADS, PSQ, SE, Opt, and Pes scores; dependent variables: SES_A or SES_S).*

|  |  | *a_1_* | *se* | *LL*  *CI* | *ULCI* | *a_2_* | | *se* | *LL*  *CI* | *UL*  *CI* | *b* | | *se* | | *LL*  *CI* | | *UL*  *CI* | *c‘* | *se* | *LL*  *CI* | *UL*  *CI* | *a_1_ a_2_b* | *se* | *LL*  *CI* | *UL*  *CI* |
| --- | --- | --- | --- | --- | --- | --- | --- | --- | --- | --- | --- | --- | --- | --- | --- | --- | --- | --- | --- | --- | --- | --- | --- | --- | --- |
| **TQ-SES_A** | |  |  |  |  |  | |  |  |  |  | |  | |  | |  |  |  |  |  |  |  |  |  |
| **ISR** | **ADS** | .02 | .00 | .02 | .02 | 10.04 | | .83 | 8.41 | 11.67 | .12 | | .04 | | .03 | | .20 | .22 | .02 | .18 | .25 | .02 | .01 | .01 | .04 |
| **DS** | **ADS** | .03 | .00 | .03 | .03 | 8.34 | | .37 | 7.61 | 9.06 | .15 | | .05 | | .05 | | .24 | .22 | .02 | .19 | .26 | .04 | .01 | .01 | .06 |
| **AS** | **ADS** | .02 | .00 | .02 | .03 | 4.53 | | .36 | 3.81 | 5.24 | .18 | | .04 | | .11 | | .25 | .22 | .02 | .18 | .26 | .02 | .00 | .01 | .03 |
|  | **PSQ** | .02 | .00 | .02 | .02 | .06 | | .01 | .05 | .07 | 6.17 | | 1.86 | | 2.53 | | 9.81 | .25 | .02 | .22 | .29 | .01 | .00 | .00 | .01 |
|  | **T** | .02 | .00 | .02 | .02 | .06 | | .01 | .05 | .08 | 4.64 | | 1.41 | | 1.88 | | 7.41 | .25 | .01 | .21 | .29 | .01 | .00 | .00 | .01 |
|  | **W** | .02 | .00 | .02 | .02 | .09 | | .01 | .08 | .10 | 4.55 | | 1.48 | | 1.64 | | 7.45 | .26 | .02 | .22 | .29 | .01 | .00 | .00 | .02 |
|  | **D** | .02 | .00 | .02 | .02 | .04 | | .01 | .02 | .06 | 2.25 | | 1.14 | | .02 | | 4.48 | .27 | .02 | .24 | .31 | .01 | .00 | .00 | .00 |
|  | **Pes** | .02 | .00 | .02 | .02 | .10 | | .03 | .05 | .14 | 1.15 | | .38 | | .41 | | 1.90 | .26 | .02 | .23 | .30 | .00 | .00 | .00 | .01 |
| **OS** | **ADS** | .02 | .00 | .01 | .02 | 4.06 | | .34 | 3.38 | 4.73 | .22 | | .03 | | .15 | | .28 | .23 | .02 | .19 | .27 | .01 | .00 | .01 | .02 |
|  | **PSQ** | .02 | .00 | .01 | .02 | .06 | | .01 | .05 | .07 | 8.02 | | 1.83 | | 4.43 | | 11.61 | .27 | .02 | .23 | .31 | .01 | .00 | .00 | .01 |
|  | **T** | .02 | .00 | .01 | .02 | .07 | | .01 | .06 | .08 | 5.71 | | 1.44 | | 2.89 | | 8.53 | .27 | .02 | .24 | .31 | .01 | .00 | .00 | .01 |
|  | **W** | .02 | .00 | .01 | .02 | .08 | | .01 | .07 | .10 | 6.24 | | 1.46 | | 3.38 | | 9.10 | .27 | .02 | .24 | .31 | .01 | .00 | .00 | .01 |
|  | **J** | .02 | .00 | .01 | .02 | -.07 | | .01 | -.08 | -.05 | -2.47 | | 1.29 | | -5.00 | | .07 | .30 | .02 | .26 | .33 | .00 | .00 | -.00 | .01 |
|  | **D** | .02 | .00 | .01 | .02 | .04 | | .01 | .03 | .06 | 2.63 | | 1.17 | | .34 | | 4.91 | .30 | .02 | .27 | .34 | .00 | .00 | .00 | .00 |
|  | **SE** | .01 | .00 | .01 | .02 | -.18 | | .02 | -.22 | -.14 | -1.08 | | .52 | | -2.09 | | -.07 | .29 | .02 | .26 | .33 | .00 | .00 | .00 | .01 |
|  | **Pes** | .01 | .00 | .01 | .02 | .10 | | .02 | .05 | .14 | 1.28 | | .39 | | .52 | | 2.05 | .29 | .02 | .25 | .32 | .00 | .00 | .00 | .00 |
| **SS** | **ADS** | .02 | .00 | .01 | .02 | 3.27 | | .43 | 2.44 | 4.11 | .20 | | .03 | | .13 | | .26 | .22 | .02 | .18 | .26 | .01 | .00 | .01 | .02 |
|  | **PSQ** | .02 | .00 | .01 | .02 | .05 | | .01 | .04 | .06 | 7.30 | | 1.76 | | 3.84 | | 10.76 | .26 | .02 | .22 | .29 | .01 | .00 | .00 | .01 |
|  | **T** | .02 | .00 | .01 | .02 | .04 | | .01 | .03 | .05 | 5.79 | | 1.36 | | 3.13 | | 8.45 | .25 | .02 | .22 | .29 | .00 | .00 | .00 | .01 |
|  | **W** | .02 | .00 | .01 | .02 | .07 | | .01 | .06 | .09 | 5.45 | | 1.42 | | 2.66 | | 8.24 | .26 | .02 | .22 | .30 | .01 | .00 | .00 | .01 |
|  | **D** | .02 | .00 | .01 | .02 | .03 | | .01 | .02 | .05 | 2.52 | | 1.13 | | .31 | | 4.73 | .28 | .02 | .25 | .32 | .00 | .00 | .00 | .00 |
|  | **SE** | .02 | .00 | .01 | .02 | -.14 | | .02 | -.19 | -.09 | -1.01 | | .51 | | -2.00 | | -.01 | .28 | .02 | .24 | .31 | .00 | .00 | .00 | .01 |
|  | **Pes** | .02 | .00 | .01 | .02 | .11 | | .03 | .06 | .16 | 1.14 | | .39 | | .38 | | 1.91 | .27 | .02 | .24 | .31 | .00 | .00 | .00 | .00 |
| **ES** | **ADS** | .01 | .00 | .00 | .01 | .97 | | .38 | .21 | .173 | .23 | | .03 | | .17 | | .29 | .23 | .02 | .19 | .27 | .00 | .00 | .00 | .00 |
|  | **PSQ** | .01 | .00 | .00 | .01 | .02 | | .01 | .01 | .03 | 9.41 | | 1.73 | | 6.02 | | 12.80 | .27 | .02 | .24 | .31 | .00 | .00 | .00 | .00 |
|  | **T** | .01 | .00 | .00 | .01 | .01 | | .01 | .00 | .03 | 7.00 | | 1.37 | | 4.32 | | 9.68 | .27 | .02 | .24 | .31 | .00 | .00 | .00 | .00 |
|  | **W** | .01 | .00 | .00 | .01 | .03 | | .01 | .02 | .05 | 7.24 | | .137 | | 4.54 | | 9.94 | .27 | .02 | .24 | .31 | .00 | .00 | .00 | .00 |
|  | **D** | .01 | .00 | .00 | .01 | .02 | | .01 | .01 | .04 | 3.10 | | 1.16 | | .82 | | 5.37 | .31 | .02 | .28 | .34 | .00 | .00 | .00 | .00 |
|  | **SE** | .01 | .00 | .00 | .01 | -.05 | | .02 | -.09 | -.00 | -1.58 | | .49 | | -2.55 | | -.61 | .30 | .02 | .27 | .33 | .00 | .00 | .00 | .00 |
|  | **Pes** | .01 | .00 | .00 | .01 | .06 | | .03 | .01 | .11 | 1.39 | | .39 | | .61 | | 2.16 | .30 | .02 | .27 | .33 | .00 | .00 | .00 | .00 |
| **TQ-SES_S** | |  |  |  |  |  |  | |  |  |  |  | |  | |  | |  |  |  |  |  |  |  |  |
| **DS** | **ADS** | .03 | .00 | .03 | .03 | 8.34 | .37 | | 7.61 | 9.06 | .07 | .03 | | .01 | | .12 | | .08 | .01 | .06 | .10 | .02 | .01 | .00 | .03 |
|  | **J** | .03 | .00 | .03 | .03 | -.14 | .01 | | -.15 | -.12 | 1.74 | .85 | | .07 | | 3.41 | | .10 | .01 | .08 | .12 | -.01 | .00 | -.01 | -.00 |
| **AS** | **ADS** | .02 | .00 | .02 | .03 | 4.53 | .36 | | 3.81 | 5.24 | .06 | .02 | | .02 | | .09 | | .08 | .01 | .06 | .10 | .01 | .00 | .00 | .01 |
|  | **W** | .02 | .00 | .02 | .02 | .09 | .01 | | .08 | .10 | 1.50 | .80 | | -.06 | | 3.07 | | .09 | .01 | .07 | .11 | .00 | .00 | -.00 | .01 |
|  | **Pes** | .02 | .00 | .02 | .02 | .10 | .03 | | .05 | .14 | .39 | .20 | | -.00 | | .79 | | .09 | .01 | .08 | .11 | .00 | .00 | .00 | .00 |
| **OS** | **ADS** | .02 | .00 | .01 | .02 | 4.06 | .34 | | 3.38 | 4.73 | .08 | .02 | | .05 | | .12 | | .09 | .01 | .07 | .11 | .01 | .00 | .00 | .01 |
|  | **PSQ** | .02 | .00 | .01 | .02 | .06 | .01 | | .05 | .07 | 3.01 | 1.04 | | .97 | | 5.05 | | .10 | .01 | .08 | .12 | .00 | .00 | .00 | .01 |
|  | **W** | .01 | .00 | .01 | .02 | .08 | .01 | | .07 | .10 | 2.55 | .83 | | .92 | | 4.18 | | .10 | .01 | .08 | .12 | .00 | .00 | .00 | .01 |
|  | **Pes** | .01 | .00 | .01 | .02 | .10 | .02 | | .05 | .14 | .47 | .21 | | .06 | | .88 | | .11 | .01 | .09 | .13 | .00 | .00 | .00 | .00 |
| **SS** | **ADS** | .02 | .00 | .01 | .02 | 3.27 | .42 | | 2.44 | 4.11 | .07 | .02 | | .04 | | .10 | | .08 | .01 | .06 | .10 | .00 | .00 | .00 | .01 |
|  | **T** | .02 | .00 | .01 | .02 | .04 | .01 | | .02 | .05 | 1.48 | .73 | | .04 | | 2.92 | | .10 | .01 | .08 | .12 | .00 | .00 | .00 | .00 |
|  | **W** | .02 | .00 | .01 | .02 | .07 | 01 | | .06 | .09 | 2.11 | .78 | | .58 | | 3.64 | | .10 | .01 | .08 | .11 | .99 | .00 | .00 | .00 |
|  | **Pes** | .02 | .00 | .01 | .02 | .11 | .03 | | .06 | .16 | .39 | 21 | | -.01 | | .80 | | .10 | .01 | .09 | .12 | .00 | .00 | .00 | .00 |
| **ES** | **ADS** | .01 | .00 | .00 | .01 | .97 | .39 | | .21 | 1.73 | .09 | .02 | | .06 | | 12 | | .08 | .01 | .06 | .10 | .00 | .00 | .00 | .00 |
|  | **T** | .01 | .00 | .00 | .01 | .01 | .01 | | .00 | .03 | 2.11 | .74 | | .67 | | 3.56 | | .11 | .01 | .09 | .13 | .00 | .00 | .00 | .00 |
|  | **W** | .02 | .00 | .09 | .15 | .03 | .01 | | .02 | .04 | 3.03 | .75 | | 1.56 | | 4.51 | | .10 | .01 | .08 | .12 | .00 | .00 | .00 | .00 |
|  | **D** | .01 | .00 | .00 | .01 | .02 | .01 | | .01 | .04 | 1.24 | .63 | | .02 | | 2.47 | | .12 | .01 | .10 | .14 | .00 | .00 | .00 | .00 |
|  | **SE** | .01 | .00 | .00 | .01 | -.05 | .02 | | -.09 | -.00 | -.62 | .29 | | -.1.20 | | -.04 | | .11 | .01 | .10 | .13 | .00 | .00 | .00 | .00 |
|  | **Pes** | .01 | .00 | .00 | 01 | .96 | .03 | | .01 | .11 | .52 | .20 | | .12 | | .92 | | .11 | .01 | .10 | .13 | .00 | .00 | .00 | .00 |

*Notes*: TQ = Tinnitus Questionnaire – German version total score, SES_A = Affective Pain Perception Scale: SES_S = Sensory Pain Perception Scale, ISR = ICD-10 Symptom Rating total score, DS = depressive syndrome, AS = anxiety-related syndrome, OS = obsessive-compulsive syndrome, SS = somatoform syndrome, ES = eating-related syndrome, ADS = Center for Epidemiological Studies Depression Scale total score, PSQ = Perceived Stress Questionnaire total score, T = tension, W = worries, J = joy, D = demands, SE = Self-efficacy Scale, Opt = Optimism Scale; Pes = Pessimism Scale, *Panels a and b:* *a* = path coefficient mediator on independent variable, *b* = dependent variable on mediator controlled for *a*, *ab* = indirect effect, se = standard error; *Panel c*: *a_1_* = path coefficient ISR (subscale) scores on independent variable, *a_2_* = path coefficient process variable (ADS, PSQ, SE, Opt, or Pes [subscale] scores) on ISR (subscale) scores, *b* = dependent variable on mediator controlled for *a_1_* and *a_2_*, c´= direct effect, *a_1_a_2_b* = serial indirect effect, se = standard error, LLCL = lower level confidence interval, ULCL = upper level confidence interval. Only significant effects are reported at *p* < .05.
