# Supplementary material for: Tinnitus-related distress and pain perceptions in patients with chronic tinnitus – Do psychological factors constitute a link?
Source: PLoS One. 2020 Jun 25;15(6):e0234807. doi: 10.1371/journal.pone.0234807 (PMC7316290; doi:10.1371/journal.pone.0234807)
Supplement: S3 Table — (DOCX) [file pone.0234807.s003.docx]

**Supplementary data - Table 3.** Simple moderation effects for paths *c*, *a* and *b.*

|  | | *Path c* | *TQ x De/Co* | *se* | *LLCI* | *ULCI* | *R^2^* | *Comp* | *se* | *LLCI* | *ULCI* | *Decomp* | *se* | *LLCI* | *ULCI* |
| --- | --- | --- | --- | --- | --- | --- | --- | --- | --- | --- | --- | --- | --- | --- | --- |
| **TQ** | | |  |  |  |  |  |  |  |  |  |  |  |  |  |
|  | | **SES_A** | .18 | .06 | .07 | .29 | .01 | .24 | .03 | .18 | .29 | .41 | .05 | .32 | .51 |
|  | | **SES_S** | .14 | .03 | .08 | .20 | .01 | .07 | .02 | .05 | .10 | .21 | .03 | .16 | .26 |
|  | *Path a* | |  |  |  |  |  |  |  |  |  |  |  |  |  |
| **TQ** | | |  |  |  |  |  |  |  |  |  |  |  |  |  |
|  | **ADS** | | .19 | .06 | .06 | .31 | .01 | .37 | .03 | .31 | .43 | .56 | .06 | .45 | .67 |
|  | **T** | | -.00 | .00 | -.01 | -.00 | .01 | .01 | .00 | .01 | .01 | .00 | .00 | .00 | .01 |
|  | **SE** | | -.01 | .00 | -.02 | -.00 | .00 | -.01 | .00 | -.02 | -.01 | -.02 | .00 | -.03 | -.02 |
|  | **Pes** | | .01 | .01 | .00 | .02 | .00 | .01 | .00 | .01 | .02 | .02 | .00 | .01 | .03 |
|  | *Path b* | |  |  |  |  |  |  |  |  |  |  |  |  |  |
|  | **SES_A** | |  |  |  |  |  |  |  |  |  |  |  |  |  |
| **AS** |  | | 1.32 | .57 | .20 | 2.43 | .00 | 2.49 | .40 | 1.70 | 3.28 | 3.80 | .40 | 3.02 | 4.59 |
|  | **SES_S** | |  |  |  |  |  |  |  |  |  |  |  |  |  |
| **ADS** |  | | .07 | .03 | .01 | .12 | .00 | .10 | .02 | .06 | .13 | .16 | .02 | .12 | .20 |
| **ISR** |  | | 1.55 | .49 | .59 | 2.50 | .01 | 2.13 | .34 | 1.47 | 2.79 | 3.68 | .35 | 2.99 | 4.36 |
| **OS** |  | | .87 | .32 | .24 | 1.51 | .01 | .57 | .22 | .15 | .99 | 1.44 | .24 | .97 | 1.91 |
| **SS** |  | | .77 | .35 | .10 | 1.45 | .00 | 1.15 | .24 | .67 | 1.62 | 1.92 | .25 | 1.44 | 2.40 |
| **ES** |  | | 1.09 | .34 | .43 | 1.75 | .01 | .50 | .22 | .07 | .94 | 1.59 | .25 | 1.10 | 2.09 |
| **Sup** |  | | 1.01 | .51 | .01 | 2.02 | .00 | 2.28 | .36 | 1.57 | 2.99 | 3.29 | .36 | 2.58 | 4.00 |

*Notes.* TQ = Tinnitus Questionnaire – German version total score, SES_A = Affective Pain Perception Scale: SES_S = Sensory Pain Perception Scale, ADS = Center for Epidemiological Studies Depression Scale total score, PSQ = Perceived Stress Questionnaire total score, T = tension, W = worries, J = joy, D = demands, ISR = ICD-10 Symptom Rating total score, DS = depressive syndrome, AS = anxiety-related syndrome, OS = obsessive-compulsive syndrome, SS = somatoform syndrome, ES = eating-related syndrome, Sup = supplementary scale, SE = Self-efficacy scale, Pes = Pessimism scale; *R^2^* = effect size for the interaction. Comp = patients with compensated tinnitus, Decomp = patients with decompensated tinnitus, De/Co = Moderator “*tinnitus-related distress level*” (decompensated vs. compensated). Only significant effects are reported at *p* < .05.
