# Supplementary material for: Tinnitus-related distress and pain perceptions in patients with chronic tinnitus – Do psychological factors constitute a link?
Source: PLoS One. 2020 Jun 25;15(6):e0234807. doi: 10.1371/journal.pone.0234807 (PMC7316290; doi:10.1371/journal.pone.0234807)
Supplement: S4 Table — (DOCX) [file pone.0234807.s004.docx]

**Supplementary data - Table 4.** Moderated mediation effects for affective and sensory pain perception.

| **TQ-SES_A** | *ab*De/Co* | *se* | *LLCI* | *ULCI* | *Comp* | *se* | *LLCI* | *ULCI* | *Decomp* | *se* | *LLCI* | *ULCI* |
| --- | --- | --- | --- | --- | --- | --- | --- | --- | --- | --- | --- | --- |
| **ADS** | .07 | .04 | .00 | .14 | .08 | .01 | .05 | .11 | .15 | .03 | .09 | .22 |
| **AS** | .06 | .03 | .01 | .12 | .03 | .01 | .02 | .05 | .09 | .03 | .01 | .12 |
| **TQ-SES_S** |  |  |  |  |  |  |  |  |  |  |  |  |
| **AS** | .03 | .02 | .00 | .07 | .02 | .00 | .01 | .03 | .05 | .02 | .02 | .08 |

*Notes.* TQ = Tinnitus Questionnaire – German version total score, SES_A = Affective Pain Perception Scale: SES_S = Sensory Pain Perception Scale, AS = anxiety syndrome; ADS = Center for Epidemiological Studies Depression Scale total score, LLCL = lower level confidence interval, ULCL = upper level confidence interval; *R^2^* = effect size for the interaction. Comp = patients with compensated tinnitus, Decomp = patients with decompensated tinnitus, De/Co = Moderator “*tinnitus-related distress level*” (decompensated vs. compensated). Only significant effects are reported at *p* < .05.
